# Hydrological dataset on mediterranean catchments of the real collobrier observatory for 1967–2024.

**DOI:** 10.1016/j.dib.2026.112811

**Published:** 2026-05-05

**Authors:** Nathalie Folton, Patrick Arnaud, Mathieu Tolsa

**Affiliations:** INRAE, Aix Marseille Univ., RECOVER, 3275 route Cézanne, 13100 Aix-en-Provence, France

**Keywords:** Measurements, Streamflow, Rainfall, Mediterranean catchments, Soil humidity, High and low flows

## Abstract

The Réal Collobrier hydrological observatory, located in south-eastern France and managed by INRAE (formerly Cemagref) since 1966, is a benchmark site for regional hydro-climatology. Created by the Ministry of Agriculture, its initial objective is to improve understanding of hydrological processes in Mediterranean regions underlain by metamorphic soils. The observatory’s catchment, situated in the Maures massif near the Mediterranean coast, is densely instrumented. Flow measurements are collected at the outlets of ten small forested nested catchments (ranging from 1.57 to 70 km²), including four headwater streams. A dense network of 15 rain gauges records rainfall data at a fine temporal scale. The dataset also includes climatological data, water temperature and soil data. The vegetation is dominated by forest communities on crystalline substrates (maquis of heath, cork oak, maritime pine, and chestnut). The geological formations are predominantly crystalline, with metamorphism increasing from east to west (from gneiss to schists and phyllites) [1]. Direct human influence has been negligible over the past 60 years, with land use and land cover remaining almost unchanged, except for a wildfire in 1990 that affected one small sub-catchment.

All data presented in this article are available in the INRAE hydrological observatory's open database (https://bdoh.inrae.fr/). The article describes the long-term dataset collected at the observatory and the validation procedures. The raw dataset underwent quality control, gap filling, and homogenization procedures to ensure temporal consistency and to improve data reliability. This rigorous quality control process results in a robust dataset used for research purposes.

This well-documented hydro-climatic information can significantly advance understanding of hydrological processes [2-6], help validate and evaluate models in Mediterranean environments [7-9]. Given the non-perennial nature of rivers in this area, these data are particularly useful for studying the origin of intermittent flow, as well as the start and end dates of flow period [10]. The hydrological dataset now spans 58 years, offring the opportunity to evaluate long-term hydrometeorological trends [11,12]. Since 2019, observations of soil moisture at several depths have been added, providing valuable information on soil water availability and vegetation dynamics.

The Réal Collobrier catchment area is part of the SOERE-RBV (Long-Term Observation and Experimentation System for Environmental Research – Mountain Basin Networks), which belongs to the OZCAR research infrastructure [13], certified by AllEnvi (National Research Alliance for the Environment) (http://www.ozcar-ri.org/real-collobrier/).

Specifications Table

**Table:** 

Subject	Earth & Environmental Sciences
Specific subject area	River discharge and temperature, precipitation, climatic measurements (air temperature, air humidity, wind intensity and direction, solar radiation) and soil measurements (humidity and temperature) on Mediterranean research site.
Type of data	Table, time series filter
Data collection	River discharge and water temperature are collected from ten hydrometric stations equipped with a hydrostatic pressure probe. Fifteen rain gauges record precipitation data using a tipping bucket rainfall. Climatic data are collected from one station with five sensors: a thermometer, an anemometer and a wind vane, an air-humidity sensor, a pyranometer. They measure air temperature and humidity, wind speed and direction, global solar radiation. Six soil humidity sensor measure soil temperature and humidity at different depths. All data are recorded by automatic acquisition systems powered by solar panel or batteries. The data are filtered to eliminate outliers and effectively reduce inhomogeneities. Each station is managed and maintained by RECOVER research unit of INRAE.
Data source location	The Real Collobrier observatory is located in South-East France, at the western end of the Maures mountain range on the Mediterranean coast. The data is managed and stored at the facilities of INRAE Aix-en-Provence, France.
Data accessibility	All datasets are archived and publicly accessible through the institutional BDOH–INRAE repository [14]: https://bdoh.inrae.fr/REAL-COLLOBRIER/ Data identification number: https://dx.doi.org/10.17180/obs.real Direct URL to data: https://dx.doi.org/10.17180/obs.real Instructions for accessing these data: Please note that downloading BDOH data requires creating an account, which is free but limited to the BDOH data policy, including the non-commercial use of data. The complete dataset is available through a link hosted on «data.gouv.fr». Access link: https://doi.org/10.57745/J2FZI5
	All datasets supporting this article are publicly available through both repositories: the institutional BDOH–INRAE repository and the public copy of the dataset archived on « data.gouv.fr ».
Related research article	Folton, N., Martin, E., Arnaud, P., L'Hermite, P., and Tolsa, M.: A 50-year analysis of hydrological trends and processes in a Mediterranean catchment, Hydrol. Earth Syst. Sci., 23, 2699–2714, https://doi.org/10.5194/hess-23-2699–2019, 2019.

## 1 Value of the Data

• The long-term hydrological measurements provide a valuable reference for analysing hydrological processes, assessing fine-scale spatio-temporal variability and studying hydro-climatic trends, climate variability, and natural cycles in Mediterranean forest environments. Due to minimal human influence, the hydrological cycle of the catchment is very close to their natural state.
• Hydrological data are widely used to calibrate and validate hydrological models and to develop new methods [15,16]. The nested structure of the instrumented catchments at the Réal Collobrier observatory are useful for the comparison of upstream and downstream hydrographs, the analysis of flood and low flow conditions, spatial downscaling, and the application of distributed hydrological modeling approaches [[7], [8], [9]]. The area has been affected by several extreme hydrometeorological events, including a once-in-a-century flood in November 2011, a record-breaking heat dome in November 2022, and an extended drought from 2020 to 2023.
• This high-quality dataset offers valuable insights for both water managers and researchers. For instance, it supports flood forecasting in the Eastern Mediterranean and contributes to water resource management studies. As a result, the hydrometric station at Pont de Fer now includes a flood monitoring and forecasting system integrated into the Flood Forecasting Service (SPC) for the Eastern Mediterranean area [17]. Data at Pont de Fer are also accessible via the Vigicrues platform (https://www.vigicrues.gouv.fr/).
• Since 2019 continuous measurements of soil moisture have been recorded including the dynamic of soil temperature at multiple depths and under two contrasting microclimates (vegetated and non-vegetated sites). The dataset aids to evaluate model water transfers in soils, to assess groundwater recharge and to monitor water stress, particularly in the context of fire risk prevention [18,19].
• A wide range of research projects have already drawn on this dataset in hydrological and multidisciplinary studies. Notable examples include the impact of vegetation on key hydrological processes, such as interception, infiltration, runoff formation and evapotranspiration [[2], [3], [6]]. Other studies have examined changes in the hydrological behavior of the Rimbaud gneissic catchment following the August 1990 wildfire, which destroyed 85% of its vegetation cover [[10], [20], [21]]. The dataset has also supported investigations into stream intermittency, exploring its origins and drivers, as well as studies of hydrological mechanisms during floods, integrating soil moisture observations [[4], [5], [10]] or natural chemical and isotopic tracing [3]. Finally, it has enabled analysis of hydrological trends over the 50-year monitoring period in the context of climate change [1,12].
• The Database for Hydrology Observatories (BDOH) provide the management, curation, and dissemination of long-term observatory data, promoting data reuse and reproducibility. It ensures long-term archiving and provides permanent access to datasets, which can be cited using a digital object identifier [14].

## 2 Background

Access to long-term and hight-quality data is essential for analysis and modeling in Mediterranean regions characterized by high precipitation variability, with an alternation of prolonged droughts and intense storms [[22], [23], [24], [25]]. Water resources are basically limited in most Mediterranean ecosystems given the long drought periods [26,27]. These conditions presents challenges in terms of water management, flood forecasting, and ecological studies, particularly in catchments with intermittent flow. In this context, long-term observation of the fate of water resources is essential to understanding key processes underway and their interaction with agricultural and land management to better assess the main environmental risks and identify the mitigation levers [22,28,29].

The observatory features a dense instrumentation network, over the period 1967 −2024, including fifteen automatic rain gauges across 70 km², flow- measurements in 10 nested sub-catchments, and continuous records of soil moisture, water temperature, and climatic variables. The dataset is compiled to provide a comprehensive, long-term record of hydrological data, which help to facilitate model design, calibration and validation [[6], [7], [8], [9],^30^,^31^]. Hydrological modeling interpret the observed dynamics and test hypotheses about the underlying mechanism [[11], [16],^20^,^24^,^25^]. It is a means for extending the significance of the results of an observatory on non gauged catchments [32,33]. High-quality long-term data allows also for research on climate impacts in Mediterranean watersheds [12,[34], [35], [36]] and for interdisciplinary research on vegetation-hydrology interactions and ecological responses [2,6,10,37].

Part of the SOERE-RBV network and OZCAR research infrastructure [13] (ozcar-ri.org/real-collobrier), the dataset ensures long-term preservation and reproducibility in line with INRAE’s open science policy.

## 3 Data Description

**Fig. 1:**
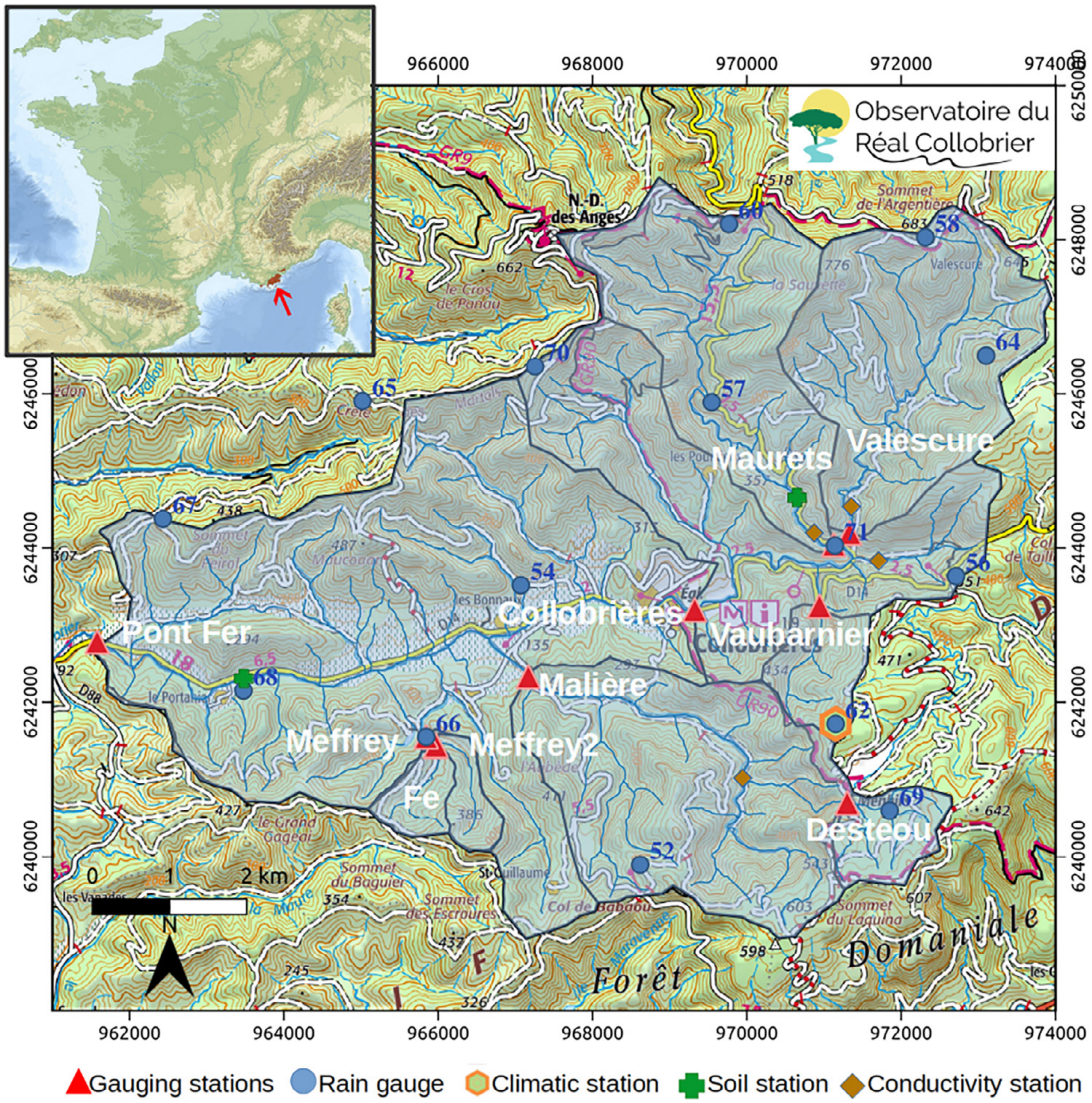
Status of the measurement network.

This section describes the hydrometeorological dataset collected by the observatory and the functionnality of the BDOH Réal Collobrier database. The dataset includes rainfall time series (Fig. 1 and Table 1) from fifteen sites, with an average spatial density is 0.21 rain gauges per km², corresponding to one station every 5 km² on average. Ten sites provide time series of water level and discharge (Fig. 1 and Table 2), and water temperature data have recently been added. Four stations measure conductivity (Table 3). One meteorological station records time series of climatic variables (Table 5), and three sites measure soil moisture at various depths and different environments (Tables 5).

**Table 1:** List and main characteristics of 15 rain gauges listed in alphabetical order with 14 stations currently in service.

Num	Name	Longitude	Latitude	Elevation (m)	Measuring period
56	Anselme	6,3526,144	43,2416,666	355	1967- 2024
52	Babaou	6,3041,160	43,2076,631	440	1967- 2024
54	Bonnaux	6,2859,792	43,2423,381	166	1967–2024
71	Bourdin	6,3372,633	43,2438,979	207	1967–2024
66	Bourjas	6,2708,898	43,2235,931	236	1967–2024
60	Fourche	6,3225,563	43,2819,226	543	1967–2024
57	Guerin	6,3163,057	43,2628,114	348	1967–2024
62	Lambert	6,3361,847	43,2231,158	542	1967–2024
64	Louviere	6,3626,380	43,2653,131	645	1967–2024
65	Martels	6,2628,289	43,2630,540	518	1967–2024
58	Mouton	6,3537,739	43,2794,092	648	1967–2024
67	Peyrol	6,2284,489	43,2525,366	320	1967–2024
68	Portanière	6,2387,459	43,2319,179	107	1967–2024
69	Rimbaud	6,3442,456	43,2126,567	554	1967–2024
70	Vaudrèches	6,2908,723	43,2689,644	487	1967–2024

**Table 2:** List and main characteristics of catchments and water level measurements.

Num	Name	River name	Area (km²)	Outlet elevation [m]	Gauging system	Monitoring period
01	Pont de Fer	Real Collobrier	70.5	90	V-notch weir	1967–2024
02	Collobrières	Real Collobrier	29	154.8	Rectangular notch weir	1970–2024
04	Malière	Malière	12.4	137.8	Bridge riffle	1967–2024
05	Valescure	Valescure	8.5	197	V-notch weir	1967–2024
06	Maurets	Maurets	9.2	195	V-notch weir	1967–2024
07	Vaubarnier	Vaubarnier	1.49	220	V-notch weir	1967–2024
08	Desteou	Rimbaud	1.53	469	V-notch weir	1967–2024
19	Meffrey	Meffrey	1.54	155	V-notch and rectangular weir	1985/2014
21	Fe	Meffrey	0.83	170	V-notch and rectangular weir	2019–2024
22	Meffrey 2	Meffrey	0.69	165	V-notch and rectangular weir	2019–2024

**Table 3:** Characteristics of conductivity sensor.

Localisation	Latitude	Longitude	Monitoring period
Cactus	43.224808	6.312762	07/2022–2024
Maurets	43.229868	6.240364	07/2022–2024
Rascas	43.250373	6.331136	05/2024–2024
Valescure	43.221706	6.337927	07/2022–2024

Data span the period of record for each site with the earliest dating from 1967 up to the last site visit in 2024. These data are updated annually. Missing date/time stamps are inserted in the time series and data are reported as missing.

All data time series and metadata are available through BDOH Réal collobrier database (https://dx.doi.org/10.17180/obs.real). The Hydrology Observatory Database (BDOH) provides a standardized framework for the management, long-term curation, and open dissemination of hydrological time series generated by long-term observatories. It ensures consistent data structuring, traceability, and accessibility to support reuse across research. BDOH is an evolving database, developed at INRAE or the needs of researchers. Although the web link leads to a French-language page, users can switch to English by clicking on the language flag located in the top right corner. Data are organized by station and include information on the data provider, the period of availability (with dates in Coordinated Universal Time, UTC) and the amount of the available data (Fig. 1A).

The database contains continuous time series measuring the following parameters:

- Rainfall in mm (time series code: PRCP) for fifteen stations (Table 1);
- Water level in cm (time series code: HT) for ten stations (Table 2);
- Discharge in m^3^s^−1^ or ls^−1^ (time series code: DEB) for ten stations, calculated using the gauging curves specific to each station (Table 2);
- Water temperature in °C for ten stations (Table 2);
- Conductivity S/cm for four stations (Table 3);
- Soil moisture in percentage (%) and soil temperature in °C on three sites with different environments observed (Table 5) and different depths;
- and climatic variables for one station including air temperature in °C, solar radiation in Jcm^−1^, wind direction in °C, wind speed in ms^−1^ and air humidity in % (Table 4).

**Table 4:** Description of instruments to measure climatic data on Lambert site.

Measure	Instrument	Type	Monitoring period
Air temperature	temperature sensor	Onset HOBO-S® -THB -M00X	2021–2024
Air humidity	relative humidity sensor	Onset HOBO-S® -THB -M00X	2021–2024
Global radiation	pyranometer	Onset HOBO-S® -LIB-M003	2021–2024
Wind speed	anemometer analogic	Onset HOBO® -S -WSB-M003	2021–2024
Wind direction	wind sensor	Onset HOBO-S® -WDA-M003	2021–2024
Barometric Pressure	barometric pressure Smart Sensor	Onset HOBO®-S-BPB-CM50	2021–2024
Soil humidity	moisture sensor	Sentek® drill and drop probe	2019–2024
Soil temperature	temperature sensor	Sentek® drill and drop probe	2019–2024

**Table 5:** Characteristics of moisture sensor.

Localisation	Num	Latitude	Longitude	Depths [cm]	Environment
Portanière	P1	43.229868	6.240364	5,15,25,35,45,50,55,65,75	Meadow,Herbaceous vegetation
Lambert	P1	43.221706	6.337927	5, 15, 25	Under tree (holm oak)
Lambert	P2	43.221698	6.337883	5, 15, 25	Meadow,Herbaceous vegetation
Maurets	P1	43.250373	6.331136	5, 15, 25	No vegetation
Maurets	P2	43.250408	6.331178	5, 15, 25	Herbaceous vegetation

**Table 6:** Quality codes used in BDOH database.

Code	Status	Description
v	Valid	The value is coherent with the other values of the parameters
a	Missing information	The quality of the data cannot be assessed
l	Missing value	Missing value, the value associated is −9999
i	Invalid	Outlier value that was removed, the value associated is −9999
d	Questionable	The value is not coherent with the other values of the parameters
e	Estimated value	The value was estimated following method described in the next section
lq	Limit of quantification	The value is lower than the limit of quantification
- and climatic variables for one station including air temperature in °C, solar radiation in Jcm^−1^, wind direction in °C, wind speed in ms^−1^ and air humidity in % (Table 4).

**Fig. 2:**
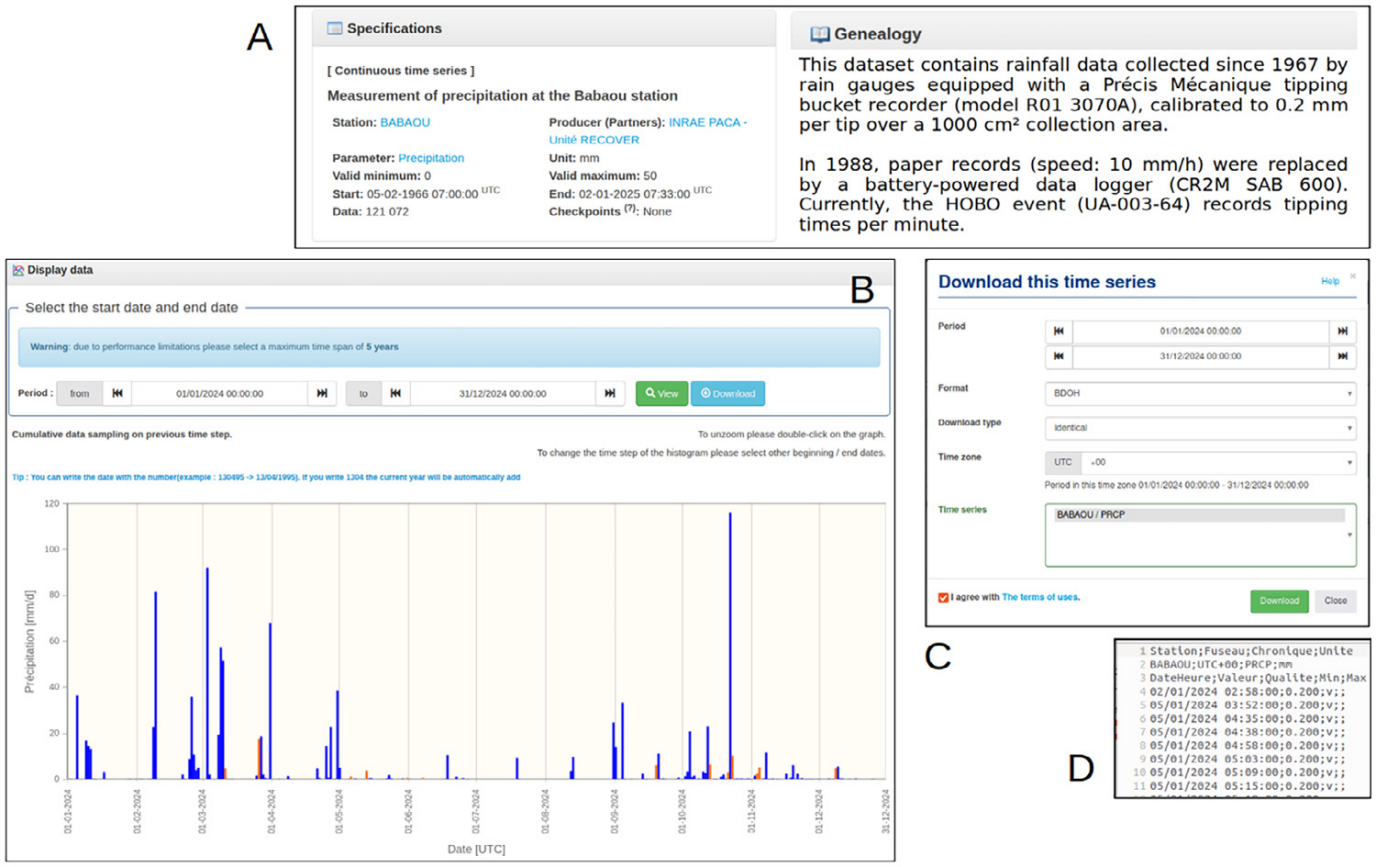
Example of time series available at the Babaou station in the BDOH Réal Collobrier database: (A) screenshot of specification rainfall time series and genealogy of the station, (B) screenshot of the time series graph, (C) screenshot of the window for choosing download parameters of the time series, (D) screenshot of the flat text file of the time series and associated quality code using gedit (v41.0).

Users can read specifications and genealogy on the station (Fig. 2A), visualize directly each time series (Fig. 2B) and download them individually after selecting several parameters (Fig. 2C) such as:

- the period of interest by selecting start and end dates;
- the file format: BDOH (raw) or Hydro2-QTVAR (a specific format used by the French national hydrological services);
- the type of transformation and time step: identical (raw data, constant or variables steps), linear interpolation at constant time steps, means (1 or 6 h, daily, monthly, yearly, event scale). This option applies exclusively to computed and continuous time series.;
- the time zone (by default in the database: UTC +00).

Data are sent by e-mail in a compressed folder (export.zip) containing the time series as flat text files saved in a comma-delimited (STATION_time series code.txt, Fig. 2D) and another files: (Report.txt) that contains all the necessary metadata (producer, parameter, genealogy, time zone), (FormatsExport.pdf) which explains the BDOH data format and the license agreement for the use of BDOH.

Any software (Excel, R etc.) can process time series files. All data are subject to rigorous quality control procedures, combining automated filters and manual validation. Each record is assigned a quality code following the BDOH standard (Hydrology Observatory Database, INRAE), indicating its reliability (Table 6).

A complementary spatial data with catchments boundaries and instruments locations is available at https://doi.org/10.57745/AVVBFJ.

## 4 Experimental Design, Materials and Methods

The section details the instrumentation network, measurement periods, acquisition protocols, and data processing steps prior to database integration.

### 4.1 Rainfall data: Sampling and measurement protocols

#### 4.1.1 Instrumentation

The rain gauge network currently comprises fourteen stations (Bonnaux station has been closed in 2020) and provides instantaneous data. Automatic tipping-bucket rain gauges (Précis Mécanique R30302 model, with a 1000 cm² collection area) were installed to monitor rainfall intensity ans its spatial pattern. Every 0.2 mm of precipitation triggers a bucket tip, recorded with a timestamp (accuracy: ±1 min). Installed in 1967, the network has undergone successive upgrades, including improvements to gauge aerodynamics in 1988 and the transition from paper records to digital data loggers. Data acquisition systems have evolved from the CR2M SAB 600® (used until 2019) to the current HOBO® Pendant event logger. The instruments are automated and operate continuously. Data are aggregated into hourly and daily time during post-processing. Fig. 2

#### 4.1.2 Validation and quality control

Monthly on-site maintenance ensures data reliability by inspecting gauges for proper drainage, structural stability, and tipping bucket functionality. The cumulative rainfall recorded by the logger is systematically compared with the volume of water collected in a 20-liter plastic container placed beneath each gauge. Data validation follows strict criteria: errors exceeding 20% result in data being marked as invalid (quality code: "i", and rain gauge is recalibrated); errors between 10 and 20% result in the data being reported as uncertain (quality code: "d"); and errors below 10% are considered valid.

#### 4.1.3 Data processing

Each rainfall event is time-stamped, allowing for the reconstruction of rainfall intensity time series. Outliers are identified through temporal comparisons with neighboring stations and spatial analysis of cumulative rainfall curves, taking into account east-west gradients and elevation effects. Synchronization errors and time lags are taken into account. Recent data are cross-validated with COMEPHORE radar estimates (Météo-France, [38]) to correct outliers, which are assigned the quality code "e"*.* The processed data including quality codes, are stored in text and CSV formats in the INRAE BDOH open database (bdoh.inrae.fr) with time-stamped precise to the minute.

### 4.2 Hydrological data: Sampling and measurement protocols

#### 4.2.1 Instrumentation

**Fig. 3:**
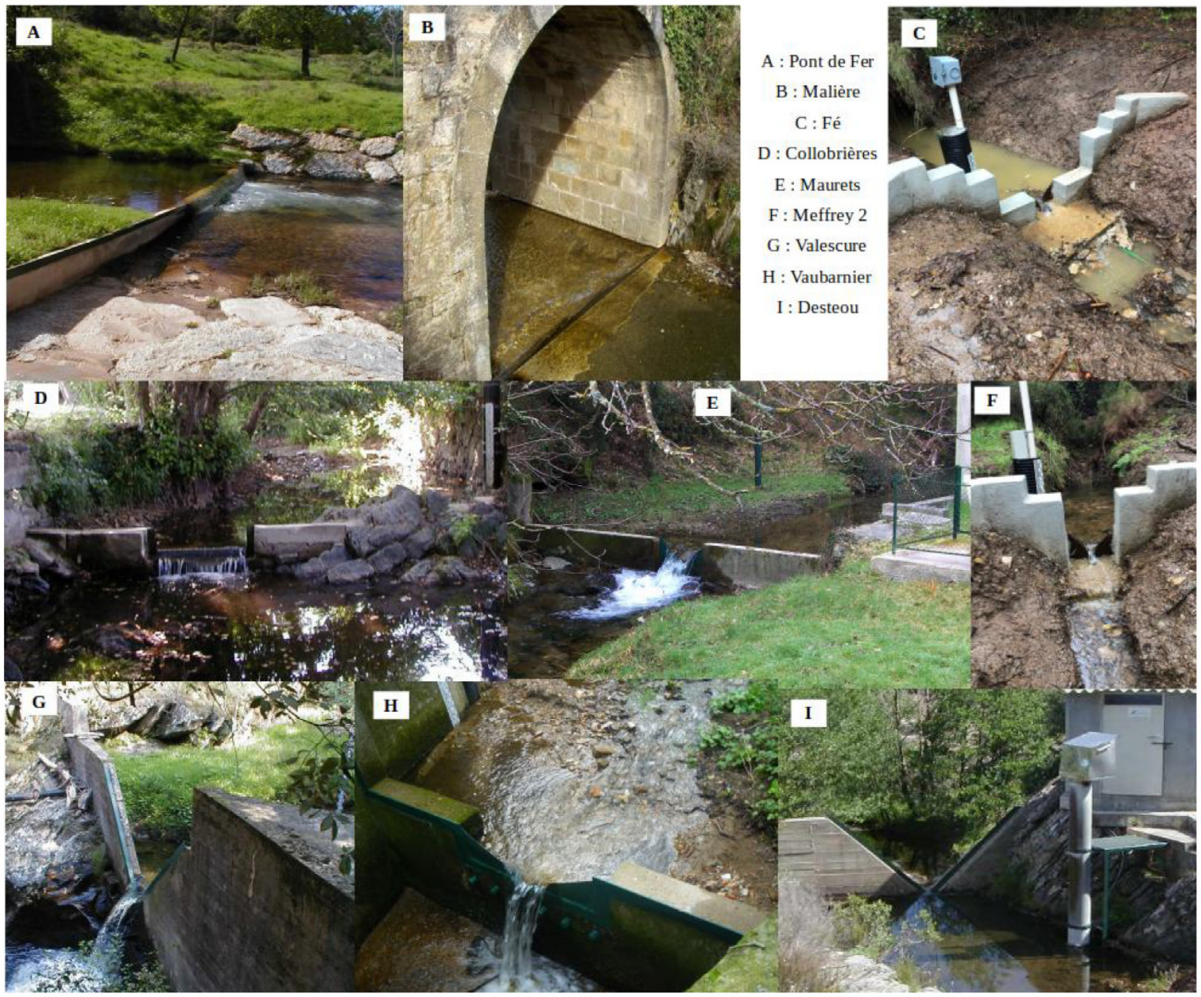
Current hydrométric stations A: Pont de Fer station, B:Malière station,C: Fé station, D:Collobrieres station, E: Mauret station, F: Meffrey2 station, G: Valescure station, H: Vaubarnier station, I: Dsteou station.

Most discharge stations are equipped with artificial controls designed to stabilize flow and enhance measurement accuracy. These include V-notch weirs for low-flow sensitivity and rectangular weirs for medium to high flows. Some stations features compund controls combining types. Photos of all currently gauging stations are provided in Fig. 3. Among them,the Malière (Fig. 3C) and Pont de Fer (Fig. 3D) stations have a single hydraulic control section.

• **Pont de Fer Station** is located near an old bridge, which facilitates access and equipment handling during flood events. The control section has maintained a flat V-shape since 1988, and a triangular weir was added in October 1992. Prior to 1988, water depth was governed by a natural section that was frequently reshaped by floods. Between 1967 and 1988, ten successive rating curves were derived from manual measurements. Since 1988 the control section has maintained a flat V-shape and a triangular weir was raised in October 1992.
• **Malière Station** is positioned adjacent to a bridge, with flow control provided by a riffle directly beneath the structure.
• **Collobrière Station** (Fig. 3D) is installed on a rocky outcrop. A rectangular discharge weir regulates medium and low flows, while high flows are governed by a natural control section.
• **The six remaining stations**—Mauret, Valescure, Vaubarnier, Desteou, Fe, Meffrey and Meffrey 2, use compound control systems. These combine a partial V-notch weir for low and medium flows with a rectangular weir for high flows, allowing for accurate definition of the gauging relationship across a wide range of discharges.

Water levels are continuously monitored at 10 stations using an evolving system of instruments. Initially, float gauges in stilling wells were recorded by a mechanical limnigraph (OTT system) on paper charts. In 1989, the system transitioned to an automatic data acquisition unit (SAB600, CR2M), and since 2018, measurements have been performed using a Bluetooth water-level recorder (MX2001) equipped with a HOBO U20 pressure sensor. This setup provides real-time barometric compensation, ensuring a water level accuracy of ±0.05% and a resolution of 0.2 cm, alongside temperature measurements with an accuracy of ±0.5 °C and a resolution of 0.1 °C (range: −20 °C to 50 °C). Data are recorded every two minutes, but if the water level change is <1 cm, the measurement is not stored to avoid redundancy. The system covers a discharge range from 0.001 m³/s to 250 m³/s, ensuring comprehensive monitoring of hydrological dynamics across varying flow conditions.

#### 4.2.2 Stage-Discharge conversion and validation

**Fig. 4:**
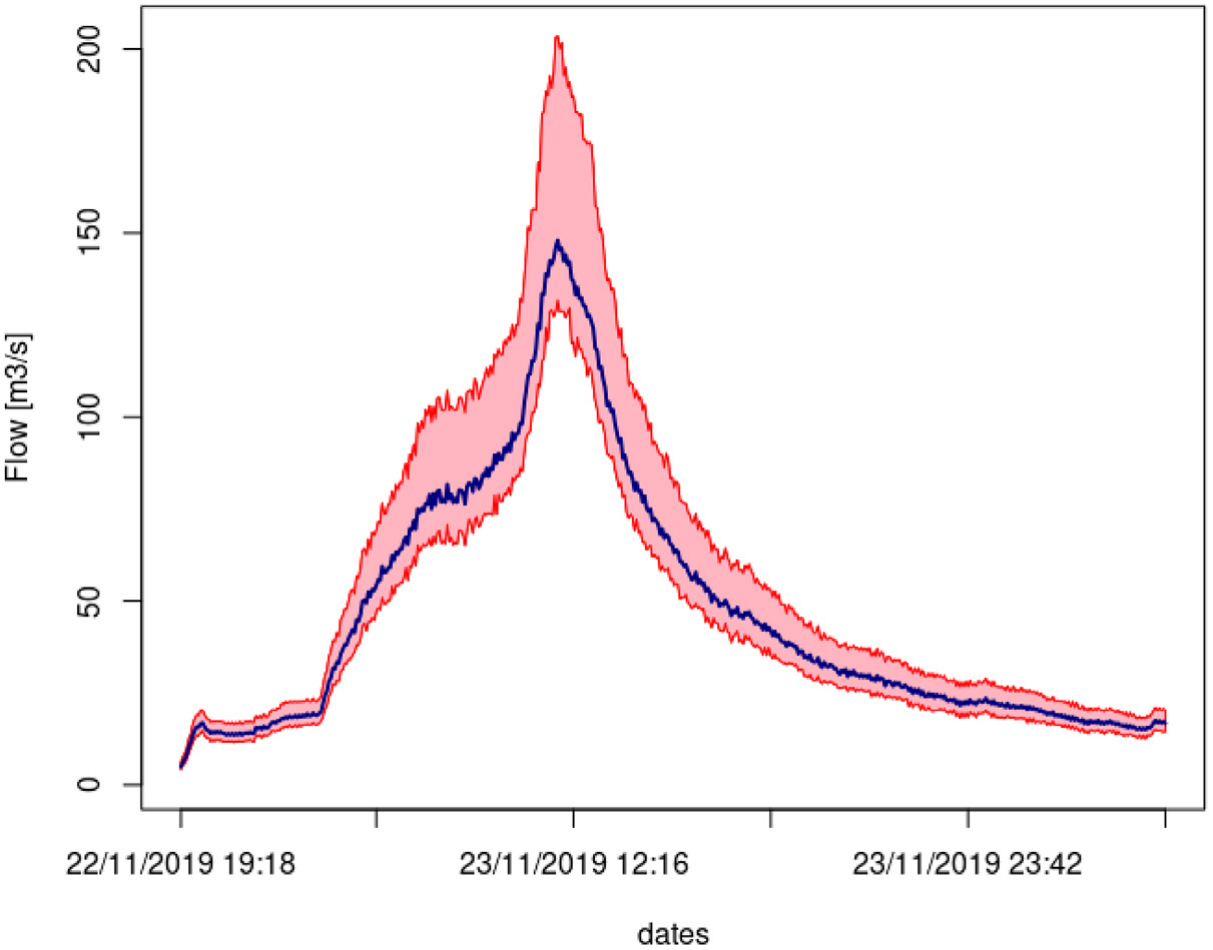
Event of 23 November 2019 on the Pont Fer catchment, hydrograph in blue and associated uncertainty represented with red shaded areas.

Water level measurements are converted to discharge values using stage-discharge rating curves developed with the BaRatin framework [39,40], which integrates hydraulic modeling and Bayesian inference to quantify uncertainties. Spot gauging measurements also supplement the calculation of these curves. As an illustration, Fig. 4 presents, for the Pont de Fer station, the hydrograph and associated uncertainties for the 23 November 2011 event. During regular field visits, visual checks of the reference graduated scales are performed during field visits to check the water level monitored. Quality control includes detecting anomalies (e.g., discontinuities, sensor drift), cross-checking time series consistency, and flagging data as "i" (invalid), "v" (valid after correction), or reliable. During zero-flow periods, temperature sensors exposed to air may yield inaccurate readings, which are systematically replaced with missing data (invalid).

#### 4.2.3 Data processing and archiving

Water levels validated are converted to discharge using rating curves, while temperature data are hourly aggregated for consistency. Processed data (including water levels, discharge, temperature and quality codes) are stored in text and CSV formats in the INRAE BDOH open database (bdoh.inrae.fr), with timestamps precise to the minute for discharge. The temperature data is aggregated on an hourly time step.

### 4.3 Conductivity data: Sampling and measurement protocols

#### 4.3.1 Instrumentation

Since summer 2022, continuous conductivity and temperature time series have been acquired across the watershed. Two gauging stations (Maurets and Valescure) are currently equipped with HOBO® U24–001 freshwater temperature-conductivity loggers, complemented by a monitoring point in the Malière sub-catchment at the Cactus site. An additional upstream station in the Rascas valley is scheduled for deployment in May 2024. The loggers provide an operating accuracy of 5 µS/cm with a 1 µS/cm resolution for conductivity, and an accuracy of 0.1 °C with a 0.01 °C resolution for water temperature. At all sites, measurements are sampled and recorded every 30 min, synchronized to the top of the hour.

#### 4.3.2 Validation and quality control

Data are retrieved on-site at least four times per year using HOBO® software during routine maintenance operations, which include a visual inspection of the site and a spot-check of conductivity using a reference meter to verify logger readings. A posteriori quality control is applied through systematic comparison with rainfall and discharge data from the closest hydrometric stations to ensure consistency in observed dynamics. When recorded conductivity values fall within the lower detection range (0–2.5 µS/cm), both conductivity and temperature measurements are replaced with missing values (quality code: "i"), as these conditions typically correspond to dry periods in these intermittent streams, which are frequent during summer.

#### 4.3.3 Data processing and archiving

Once validated, temperature and conductivity data are aggregated to hourly means to ensure consistency across the time series. Processed data are archived in text and CSV formats in the INRAE BDOH open database (bdoh.inrae.fr), alongside quality codes and metadata for full traceability.

### 4.4 Climatic data : Sampling and measurement protocols

#### 4.4.1 Instrumentation

**Fig. 5:**
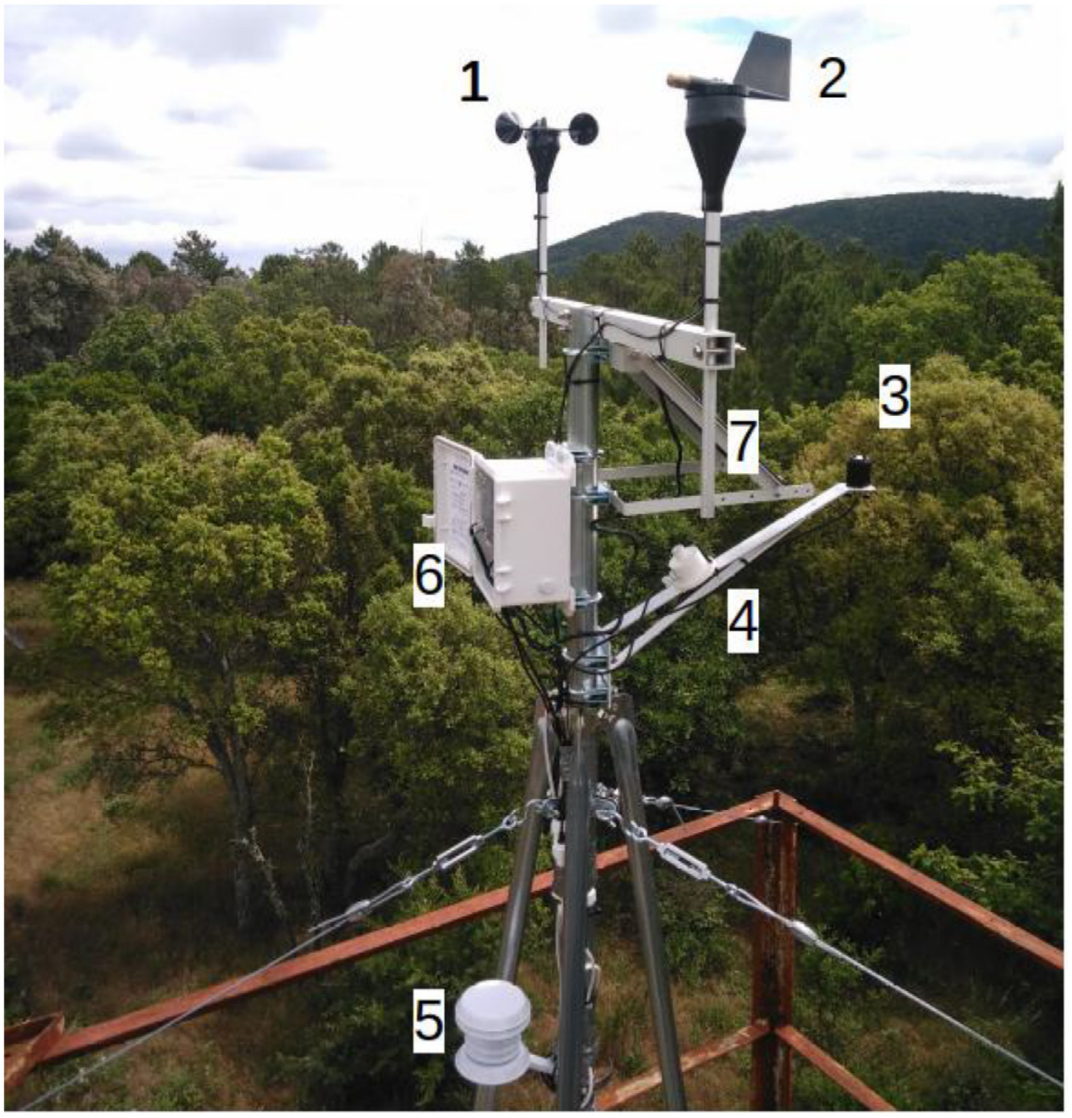
Climatic Lambert station: 1: speed wind measurement, 2: wind direction measurement, 3: global radiation measurement; 4: pression measurements, 5: air temperature and humidity, 6: central acquisition and 7: solar panel.

The climatic station records key atmospheric variables at hourly intervals, including air temperature and humidity (Onset HOBO® S-THB-M00X), global solar radiation (Onset HOBO® S-LIB-M003), wind speed (Onset HOBO® S-WSB-M003), wind direction (Onset HOBO® S-WDA-M003), and barometric pressure (Onset HOBO® S-BPB-CM50). All measurements are standardized at a reference height of two meters above ground level. The station operates autonomously, powered by solar panels and batteries, and transmits data remotely via GSM network to ensure continuous and real-time data acquisition (Fig. 5).

#### 4.4.2 Validation and quality control

Once downloaded, climatic time series undergo a rigorous quality assurance and quality control process. An R-based script identifies data gaps by detecting missing date/time stamps, which are then marked in the dataset (quality code: "l"). Outliers and values outside the specified operating range for each sensor are detected and filtered (quality code: "i"). Additionally, variables are analyzed on a daily basis using statistical distributions (mean and standard deviation) to identify and correct anomalies or inconsistent measurements (quality code: "i").

#### 4.4.3 Data processing and archiving

After validation, climatic data are reformatted for consistency and archived with quality codes and metadata. Processed datasets, including all atmospheric variables, are stored in text and CSV formats in the INRAE BDOH open database (bdoh.inrae.fr), with hourly step time to facilitate further analysis and reproducibility.

### 4.5 Soil data: sampling and measurement protocols

#### 4.5.1 Instrumentation

**Fig. 6:**
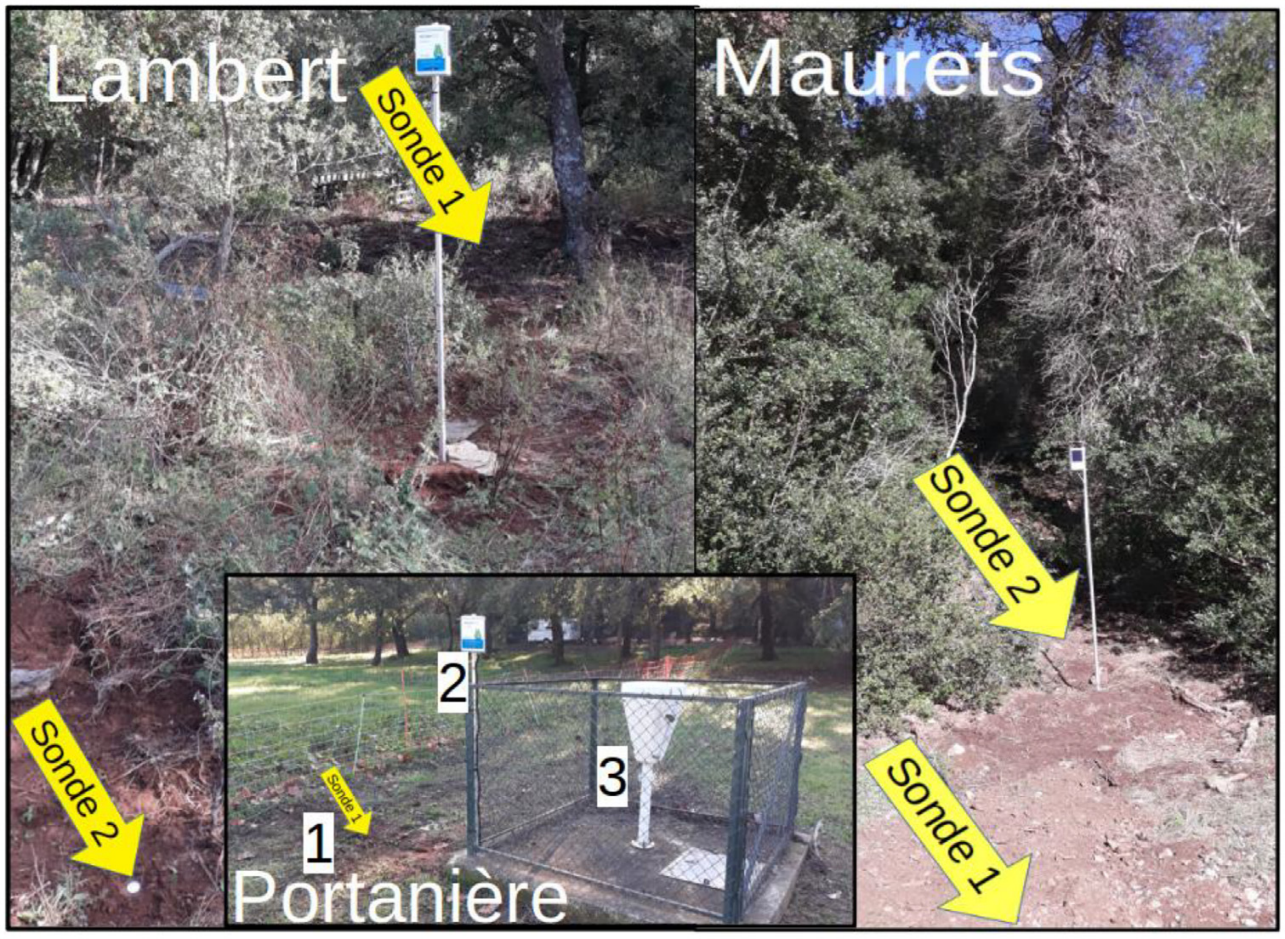
Soil station on three sites Lambert, Maurets and Portanière with 1: probe, 2: central acquisition box and solar pannel, 3: rain gauge.

Since 2019, five Sentek Drill & Drop® soil probes have been installed across three sites to monitor soil moisture and temperature. These capacitive probes measure the soil’s dielectric constant, which is directly correlated with water content, providing instantaneous soil moisture values expressed in millimeters of water. Each probe is equipped with moisture and temperature sensors every 10 cm, allowing for high-resolution vertical profiling. The network includes four 30-cm depth probes and one 90-cm depth probe, selected based on local soil characteristics. Measurements are collected and transmitted every 20 min at depths ranging from 5 cm to 85 cm (for the 90-cm probe). Data are transmitted via LoRaWAN to the Agralis® server, enabling real-time monitoring of soil conditions under contrasting microclimates. The system is autonomous and operate continuously powered by a solar panel. The probes are distributed on three sites (Fig. 6):

• Lambert site: Two 30-cm probes, one under tree cover (P1) and one in open ground (P2), spaced 3.5 m apart.
• Portanière site: One 90-cm probe.
• Maurets site: Two 30-cm probes positioned along a slope transect, one under tree cover and one in open ground.

#### 4.5.2 Validation and quality control

Monthly on-site maintenance includes visual inspections, cleaning of the data logging system, checking connections, and verifying instantaneous values to ensure data reliability. Once downloaded, soil moisture and temperature time series are aggregated into hourly time steps and undergo quality assurance and control using semi-automated R scripts. This process includes the identification and treatment of failure periods and threshold exceedances, a cross-comparison of measurements within the soil water profile and a cross-referencing with rainfall data to validate the consistency of soil moisture dynamics.

#### 4.5.3 Data processing and archiving

After validation, processed datasets, including quality indicators and metadata, are archived in text and CSV formats in the INRAE BDOH open database (bdoh.inrae.fr), with hourly time steps to support further analysis and reproducibility.

## Limitations

Long-term data present several limitations. The recording system was upgraded in 1989 with the introduction of digital recorders, improving temporal accuracy to the minute but also introducing heterogeneity in data collection, particularly for time intervals shorter than 10 min. While these upgrades enhanced data continuity and synchronization, they also created challenges in comparing pre- and post-1989 datasets.

At the Rimbaud rain gauge site, a break in record homogeneity occurred due to a metrological issue. The complete destruction of vegetation following the August 1990 fire resulted in a deficit in data capture, affecting the representativeness of measurements during this period.

Water temperature and conductivity data, even after filtering for zero-flow conditions, should be interpreted with caution. Streams in the area are intermittent, and sensors are more prone to malfunction during periods of very low water levels, potentially leading to gaps or inaccuracies.

Low-flow discharge measurements are particularly sensitive to errors, as they rely on estimating very small water depths. Under such conditions, even minor inaccuracies in water-level readings can introduce significant uncertainties in calculated discharge values.

Climatic data may present inconsistencies due to sensor malfunctions, such as a dirty pyranometer between monthly maintenance visits, which can introduce undetected biases in solar radiation measurements. While quality control filters out obvious outliers, subtle drifts (e.g., in humidity or wind speed sensors) may affect long-term comparability. Additionally, extreme weather events (e.g., storms or heatwaves) can temporarily exceed sensor operating ranges, leading to data gaps or artifacts. The autonomous GSM transmission system, while robust, is not immune to connectivity issues, particularly in remote areas.

## Ethics Statement

The authors have read and follow the ethical requirements for publication in Data in Brief and confirm that the current work does not involve human subjects, animal experiments, or any data collected from social media platforms.

## CRediT authorship contribution statement

**Nathalie Folton:** Data curation, Validation, Conceptualization, Methodology, Formal analysis, Investigation, Software, Visualization, Supervision, Writing – original draft, Writing – review & editing, Project administration. **Patrick Arnaud:** Software, Investigation, Funding acquisition, Project administration, Writing – review & editing. **Mathieu Tolsa:** Data curation, Visualization, Writing – review & editing.
